# The Passing of Sectarianism in Medicine

**Published:** 1901-11

**Authors:** 


### The Passing of Sectarianism in Medicine.

UNDER this title the *St. Paul Medical Journal*, October, 1901, makes so clear and potent an argument that we quote its words in full, which we feel sure will interest every physician who favors a unification of the profession:

We are optimistic enough to believe that the conditions outlined in the closing paragraph of Dr. Reed's eloquent presidential address, delivered at the St. Paul meeting of the American Medical Association, will be brought to pass before another generation has come and gone. There is a very considerable number of able and scholarly practitioners of medicine who are at the present time barred from the privilege of professional association with those who form the great body of scientific practitioners because they were misguided and ill-advised when they began the study of medicine and the sectarian atmosphere with which they were then surrounded has clung to them ever since. Some voluntarily cling to the name which they have assumed, not because they believe in any of the absurd theories which were held when the school to which they belong was born, but simply because there is money in it. The majority, we believe, cling to the name because under the present conditions they see no way of escape and if they should openly renounce the creed which they have long since ceased to believe, they would find themselves adrift with no port in sight.

The Illinois State Medical Society, at its last meeting, by passing the following resolution, took a step which if it is endorsed by the profession at large, and if it is followed by other state societies, as we believe it will be, will do more to banish schismatic medicine than anything which has ever been done:

*Resolved*, That the school of graduation shall be no bar to membership in the Illinois State Medical Society, providing such physician is recognised by the local societies as qualified and not claiming to practise any exclusive system of medicine.

We believe that it will be generally admitted that many of those who practice medicine under some sectarian name are men of good education and are perfectly competent to take care of their patients, and indeed that they treat their patients exactly as other physicians do, and are perfectly familiar with the modern principles of pathology, diagnosis and treatment. These men, or at least a large proportion of them would be glad to drop their exclusive name and join the ranks of scientific practitioners of medicine if an opportunity to do so were offered them. A great deal of opposition would have to be overcome, especially among the older men, many of whom would find it impossible to give up the prejudices of a lifetime, but the complete organisation of the whole profession which would ultimately result from the disappearance of all the false systems which, even although they exist in name only, are still a very real barrier to the progress of scientific medicine, would accomplish such a vast amount of good that we hope to live to see it brought to pass.

---

SUBSTITUTION in physicians's prescriptions is a perennial subject for discussion in the medical journals. Just now almost every magazine is making some reference to it, stimulated to do so we presume through the letters and circulars issued by many of the manufacturing pharmacists throughout the country. It is all very well to discuss this matter from the sentimental viewpoint, but it will have quite as much effect as Mrs. Partington's attempt to mop the Atlantic ocean dry from her doorstep. The only effectual way to deal with offending druggists is through the enactment of laws prescribing adequate punishment for such serious and hazardous offenses. A law such as that recently enacted by the General Assembly of the State of Tennessee would meet the case. It is entitled an act to prevent the substitution of any drug in filling physicians's prescriptions by druggists in that state, making violation a misdemeanor and upon conviction the druggist shall be fined not less than $25 nor more than $100 for each and every offense.

---

THE Chicago Medical Society tendered a banquet to N. S. Davis, the oldest living president of the society at the Auditorium Hotel, Saturday evening, October 5, 1901. The banquet was attended by a large number of physicians, many of whom were from distant cities.

The Virchow anniversary, commemorating the eightieth birthday of the Berlin savant, was a conspicuous gathering of noted men in all parts of the world. In this country it was celebrated by a dinner given at Sherry's, New York City, Saturday evening, October 12, 1901. One of the principal speakers was Dr. Abraham Jacobi, of New York, whose own anniversary was similarly celebrated about eighteen months ago. Dr. Jacobi was most felicitous in his remarks at the Virchow dinner and added to his renown as a gifted speaker and a physician of great knowledge in medical affairs.

Drs. Osler and Welch, of Baltimore, contributed largely to the enjoyment of the occasion, which was every way a credit to its projectors.

